# A Retrospective Study of Breast Imaging Reporting and Data System and Histopathological Correlation in Breast Lumps: A Single-Center Experience at R.L. Jalappa Hospital, Kolar, India

**DOI:** 10.7759/cureus.105247

**Published:** 2026-03-14

**Authors:** Vardhan H.R, Shashirekha C A

**Affiliations:** 1 General Surgery, Sri Devaraj Urs Medical College, Sri Devaraj Urs Academy of Higher Education and Research, Kolar, IND

**Keywords:** bi-rads, breast cancer, correlation, histopathology, india, mammography, retrospective study, ultrasonography

## Abstract

Introduction: The Breast Imaging-Reporting and Data System (BI-RADS) serves as a standardized classification system for breast imaging interpretation and management recommendations. This study evaluates the correlation between BI-RADS categories and histopathological findings in patients presenting with breast lumps at a tertiary care center in India.

Methods: A retrospective observational study was conducted at R.L. Jalappa Hospital and Research Centre, Kolar, from 1st November 2024 to 30th November 2025. Female patients aged 22-78 years who underwent breast imaging with BI-RADS categorization (categories 3-5) followed by histopathological examination were included. Statistical analysis was performed using chi-square tests with calculation of sensitivity, specificity, positive predictive value (PPV), negative predictive value (NPV), and diagnostic accuracy.

Results: A total of 215 patients were analyzed with a mean age of 48.7 ± 13.2 years. BI-RADS category distribution was: BI-RADS 3 (45 cases, 20.9%), 4A (52 cases, 24.2%), 4B (41 cases, 19.1%), 4C (28 cases, 13.0%), and 5 (49 cases, 22.8%). Malignancy rates showed progressive increase: BI-RADS 3 (8.9%), 4A (32.7%), 4B (46.3%), 4C (82.1%), and 5 (91.8%). Overall diagnostic performance showed sensitivity 96.3%, specificity 38.3%, PPV 61.2%, NPV 91.1%, and accuracy 67.4%. Strong positive correlation was found between BI-RADS categories and malignancy rates (p < 0.001, X² = 36.849).

Conclusion: BI-RADS classification demonstrates strong correlation with histopathological malignancy rates in breast lump evaluation. Higher BI-RADS categories are reliably associated with increased malignancy probability, supporting the clinical utility of BI-RADS in breast lesion management and biopsy decision-making in the Indian healthcare context.

## Introduction

Breast cancer remains the most common malignancy among women globally, with an estimated 2.3 million new cases annually and a significant mortality burden, particularly in low- and middle-income countries where late-stage diagnosis is common [1,2]. Early detection through standardized imaging interpretation is crucial for improved survival outcomes and optimal patient management. 

The Breast Imaging-Reporting and Data System (BI-RADS), developed by the American College of Radiology (ACR), provides a standardized lexicon and risk stratification system for breast imaging interpretation across mammography, ultrasonography, and magnetic resonance imaging modalities [3]. This classification system has revolutionized breast imaging practice by providing consistent terminology, risk assessment, and management recommendations. 

BI-RADS classification 

The BI-RADS classification categorizes breast lesions from 0 to 6, with specific malignancy risk probabilities and evidence-based management recommendations. Category 3 lesions are considered probably benign with <2% malignancy risk, requiring short-term follow-up at six-month intervals. Category 4 lesions are suspicious for malignancy with 2-95% cancer probability and are subdivided into 4A (2-10% malignancy risk), 4B (10-50% risk), and 4C (50-95% risk) based on the degree of suspicion. Category 5 lesions are highly suggestive of malignancy with >95% probability, requiring immediate tissue sampling [4]. 

Literature review and current evidence 

Recent international studies have demonstrated varying correlations between BI-RADS categories and histopathological outcomes across different populations and healthcare settings. Ciurescu et al. (2025) reported 91% malignancy rate for BI RADS 5 lesions with an overall sensitivity of 93.8% and specificity of 80.0% in a Romanian cohort [5]. Khan et al. (2023) found 44.9% positive predictive value for BI-RADS 4 lesions in a Pakistani population [6]. 

Studies from Indian healthcare institutions have shown some variation from Western data. Aziz et al. (2022) reported 94.48% sensitivity and 63.9% positive predictive value (PPV) in a Malaysian study of 316 patients [7]. Dariya et al. (2022) found 91.67% sensitivity and 84.62% PPV in an Indian observational study [8]. These variations emphasize the importance of population-specific validation studies. 

Study rationale and institutional context 

R.L. Jalappa Hospital and Research Centre, a 1100-bed tertiary care teaching hospital affiliated with Sri Devaraj Urs Medical College, serves rural and urban populations of Kolar district, Karnataka, and neighboring states. The hospital provides comprehensive breast imaging services including digital mammography, ultrasonography, tomosynthesis, and selective MRI with established surgical oncology and pathology departments. 

Limited data exists from Indian tertiary care centers, particularly those serving diverse socioeconomic populations in rural settings. This study aims to evaluate the correlation between BI-RADS categories and histopathological findings in our institutional setting, determine diagnostic performance parameters, and validate BI-RADS utility in the Indian healthcare context. 

Study objectives 

The primary objective is to quantitatively evaluate the correlation between BI-RADS ultrasound/mammography categories (3, 4A, 4B, 4C, and 5) and final histopathological diagnosis (benign vs. malignant) in adult women presenting with breast lumps at a tertiary care center in South India.

The secondary objectives are (i) to determine diagnostic performance parameters (sensitivity, specificity, PPV, negative predictive value (NPV), accuracy), (ii) to analyze age-related patterns of breast lesion malignancy, (iii) to compare findings with published international and Indian literature, and (iv) to validate BI-RADS clinical utility in tertiary care settings.

## Materials and methods

Study design and setting

This retrospective observational study was conducted at the Departments of Radiology, Surgery, Surgical Oncology, and Pathology, R.L. Jalappa Hospital and Research Centre, Kolar, Karnataka, India. The study was approved by the Institutional Ethics Committee and conducted in accordance with the Declaration of Helsinki and institutional guidelines. 

Study population 

Female patients who presented with breast lumps between November 2024 and November 2025 were retrospectively analyzed. The study period was selected to ensure adequate case volume and recent adherence to BI-RADS 5th edition criteria. 

Inclusion criteria

The study included female patients aged 18 to 80 years who presented with palpable breast lumps and underwent breast imaging with BI-RADS categorization (categories 3, 4, and 5). Both hospitalized patients and outpatients presenting to the general surgery, surgical oncology, and gynecology outpatient departments during the study period, with complete medical records including imaging reports, biopsy results, and clinical documentation, were included. Only those with histopathological confirmation through core needle biopsy or surgical excision were included, ensuring definitive tissue diagnosis. Patients were required to have complete medical records and imaging reports available for review, and for BI-RADS category 3 lesions, a minimum six-month follow-up was mandated to confirm the benign nature of the lesion. 

Exclusion criteria 

Exclusion criteria consisted of patients with BI-RADS categories 0, 1, 2, or 6, inadequate tissue samples for histopathological diagnosis, prior history of breast cancer or ongoing treatment, incomplete documentation or imaging reports, those lost to follow-up, and male patients, as their epidemiological patterns differ significantly from women in breast lump evaluation.

Sample size calculation 

Recent international studies have demonstrated varying correlations between BI-RADS categories and histopathological outcomes. Ciurescu et al. (2025) reported 91% malignancy rate for BI-RADS 5 lesions with an overall sensitivity of 93.8% and specificity of 80.0% in a Romanian cohort [5]. Based on previous studies showing 25-35% prevalence of malignancy in BI-RADS category 4 and 5 lesions, with 95% confidence interval (CI) and 5% margin of error, the calculated minimum sample size was 196 patients using the formula: n = Z²p(1-p)/d², where Z = 1.96, p = 0.30, d = 0.05 [9]. Accounting for potential data loss and exclusions, 230 patients were initially identified, with 215 meeting the final inclusion criteria.

Imaging techniques

*Digital Mammography, Breast Ultrasonography, Digital Breast Tomosynthesis (DBT), and Magnetic Resonance Imaging Image Interpretation* 

All imaging studies were interpreted by qualified radiologists with subspecialty training in breast imaging and a minimum of five years of experience. Interpretation followed BI-RADS 5th edition criteria with standardized reporting format. Discordant cases were reviewed in multidisciplinary team meetings, including radiologists, surgeons, and pathologists. 

Histopathological examination 

Tissue Sampling

Core needle biopsies were performed under ultrasound guidance using 14-gauge automated biopsy needles. A minimum of five samples were obtained per lesion. Surgical excision was performed when core biopsy was not feasible or showed high-risk lesions requiring complete excision. 

Histopathological Processing

All specimens were processed according to standard protocols using formalin fixation and paraffin embedding. Sections were stained with hematoxylin and eosin (H&E). Immunohistochemistry was performed when indicated for subtype classification and differential diagnosis. 

Pathological Review

All cases were reviewed by qualified pathologists with expertise in breast pathology. Discordant imaging-pathology correlations were discussed in multidisciplinary meetings for consensus diagnosis. 

Data collection 

All patients in this study underwent systematic sequential evaluation following a standardized clinical pathway: (i) initial clinical presentation with breast lump, (ii) comprehensive imaging evaluation (mammography and ultrasonography) with BI-RADS categorization by a breast radiologist, (iii) multidisciplinary discussion for lesions categorized as BI-RADS 3-5, (iv) ultrasound-guided core needle biopsy or surgical excision for tissue diagnosis, and (v) histopathological examination with final diagnosis. This sequential approach ensured that imaging interpretation preceded and informed, but was not influenced by, histopathological findings. These were extracted from electronic medical records and Picture Archiving and Communication System (PACS) using a standardized data collection form. Data quality was ensured through double verification by two independent investigators. 

Statistical analysis 

Statistical analysis was performed using IBM SPSS Statistics for Windows, Version 28 (Released 2021; IBM Corp., Armonk, New York, United States) and Python 3.12.4 with NumPy, SciPy, and Pandas libraries. Descriptive statistics were calculated for all variables. Categorical variables were presented as frequencies and percentages, while continuous variables were expressed as mean Â± standard deviation. Cross-tabulation analysis was performed to assess correlation between BI-RADS categories and histopathological findings. The Chi-square test was used to determine statistical significance of associations, with p-value <0.05 considered statistically significant. Diagnostic performance metrics were calculated considering BI-RADS categories 4 and 5 as positive tests for malignancy prediction. Sensitivity, specificity, PPV, NPV, and diagnostic accuracy were calculated with 95% CIs. The odds ratio was calculated to quantify the association strength.

## Results

Patient demographics 

A total of 215 patients met the inclusion criteria and were included in the final analysis. The mean age was 48.7 ± 13.2 years (range: 22-78 years). Table 1 presents the detailed age-wise distribution of patients, showing that the majority of patients (51.1%) were in the 40-60 years age group, consistent with peak breast cancer incidence. Age distribution is as follows: <30 years (32 patients, 14.9%), 30-40 years (51 patients, 23.7%), 40-50 years (68 patients, 31.6%), 50-60 years (42 patients, 19.5%), and >60 years (22 patients, 10.2%).

**Table 1:** Patient Demographics and Age-Wise Distribution

Age Group	Number of Patients	Percentage	Benign Cases	Malignant Cases	Malignancy Rate (%)
<30 years	32	14.9	29	3	9.4
30-40 years	51	23.7	43	8	15.7
40-50 years	68	31.6	48	20	29.4
50-60 years	42	19.5	28	14	33.3
>60 years	22	10.2	14	8	36.4
Total	215	100	162	53	24.7

BI-RADS category distribution 

BI-RADS category distribution was as follows: BI-RADS 3 (45 cases, 20.9%), BI-RADS 4A (52 cases, 24.2%), BI-RADS 4B (41 cases, 19.1%), BI-RADS 4C (28 cases, 13.0%), and BI-RADS 5 (49 cases, 22.8%). Combined BI-RADS 4 and 5 categories constituted 170 cases (79.1%) of the study population, reflecting the referral nature of our tertiary care institution. 

Histopathological findings 

As shown in Table 2, 107 cases were benign and 108 cases were malignant. Among benign lesions, fibroadenoma was the most common (43 cases, 20.0% of total), followed by fibrocystic disease (28 cases, 13.0%), papilloma (12 cases, 5.6%), and phyllodes tumor (eight cases, 3.7%). 

**Table 2:** Histopathological Distribution of Breast Lesions

Histopathological Type	Number of Cases	Percentage	Classification
Fibroadenoma	43	20	Benign
Fibrocystic Disease	28	13	Benign
Invasive Ductal Carcinoma	82	38.1	Malignant
Papilloma	12	5.6	Benign
Phyllodes Tumor	8	3.7	Benign
Ductal Carcinoma In Situ	15	7	Malignant
Invasive Lobular Carcinoma	8	3.7	Malignant
Inflammatory Carcinoma	3	1.4	Malignant
Others	16	7.4	Mixed
Total	215	100	

As shown in Table 2, malignant lesions were predominantly invasive ductal carcinoma (38 cases, 17.7% of total cases), followed by ductal carcinoma in situ (seven cases, 3.3%), invasive lobular carcinoma (four cases, 1.9%), and inflammatory carcinoma (three cases, 1.4%). Four cases of invasive lobular carcinoma were identified. 

BI-RADS and histopathological correlation 

Strong positive correlation was observed between BI-RADS categories and malignancy rates, demonstrating progressive increase across categories. The detailed correlation is presented in Table 3. 

**Table 3:** BI-RADS Category Distribution and Histopathological Correlation PPV: Positive predictive value; BI-RADS: Breast Imaging-Reporting and Data System

BI-RADS Category	Total Cases	Percentage	Benign Cases	Malignant Cases	Malignancy Rate (%)	PPV (%)
BI-RADS 3	45	20.9	41	4	8.9	8.9
BI-RADS 4A	52	24.2	35	17	32.7	32.7
BI-RADS 4B	41	19.1	22	19	46.3	46.3
BI-RADS 4C	28	13.0	5	23	82.1	82.1
BI-RADS 5	49	22.8	4	45	91.8	91.8
Total	215	100	162	53	24.7	

The correlation between BI-RADS categories and histopathological findings was statistically significant (Ï‡Â² = 36.849, degrees of freedom = 4, p < 0.001), demonstrating a strong association between imaging assessment and tissue diagnosis. 

Diagnostic performance analysis 

The diagnostic performance of BI-RADS categorization was evaluated using a standard 2×2 contingency table, comparing the imaging-based classification (test result) against histopathological diagnosis (gold standard). BI-RADS categories 4 and 5 were considered positive tests for malignancy, while BI-RADS category 3 was considered a negative test. Among the 215 patients analyzed, 104 were true positives (malignant lesions correctly classified as BI-RADS 4 or 5), and 66 were false positives (benign lesions incorrectly classified as BI-RADS 4 or 5). Additionally, 41 were true negatives (benign lesions correctly classified as BI-RADS 3), and 4 were false negatives (malignant lesions incorrectly classified as BI-RADS 3). Based on this contingency table, the total number of malignant cases confirmed by histopathology was 108 (104 true positives + 4 false negatives), and the total number of benign cases was 107 (66 false positives + 41 true negatives). From these values, the diagnostic performance parameters were calculated and are presented in Table 4. The analysis demonstrated high sensitivity of 96.3% (95% CI: 90.8-99.4%) and NPV of 91.1% (95% CI: 78.8-97.5%), with moderate specificity of 38.3% (95% CI: 28.1-49.5%) and overall diagnostic accuracy of 67.4% (95% CI: 60.8-73.4%). The odds ratio of 16.2 (95% CI: 4.3-52.8) indicates a strong association between BI-RADS 4-5 categorization and malignancy.

**Table 4:** Diagnostic Performance Analysis

Statistical Measure	Value	95% Confidence Interval
Sensitivity (%)	96.3	90.8-99.4
Specificity (%)	38.3	28.1-49.5
Positive Predictive Value (%)	61.2	54.1-67.9
Negative Predictive Value (%)	91.1	78.8-97.5
Diagnostic Accuracy (%)	67.4	60.8-73.4
Odds Ratio	16.2	4.3-52.8

Age-related analysis 

Malignancy rates increased significantly with advancing age, showing a clear age-related progression. The trend was statistically significant (p = 0.001) using the chi-square test for trend, consistent with established epidemiological patterns of breast cancer incidence. 

Imaging modality utilization 

All patients (100%) underwent combined digital mammography and breast ultrasonography as standard protocol. Digital breast tomosynthesis was utilized in 89 cases (41.4%) primarily for dense breast evaluation and architectural distortion assessment. Selective contrast-enhanced MRI was performed in 23 cases (10.7%) for preoperative staging, DCIS extent assessment, and evaluation of suspected multifocal/multicentric disease. 

## Discussion

Principal findings 

This study demonstrates a strong positive correlation between BI-RADS categories and histopathological malignancy rates in breast lump evaluation at a tertiary care center in India. The progressive increase in malignancy rates from BI-RADS 3 (8.9%) to BI-RADS 5 (91.8%) validates the clinical utility and risk stratification capability of BI-RADS classification in our healthcare setting. 

BI-RADS category-specific analysis 

 *BI-RADS 3 Lesions* 

The malignancy rate for BI-RADS 3 lesions (8.9%) exceeded the ACR-recommended <2% threshold, consistent with findings from other Indian studies [10,11]. This elevation may reflect several factors including population genetics, hormonal factors, or interpretation variations. The finding suggests the need for careful evaluation and potentially shorter follow-up intervals for BI-RADS 3 lesions in our population. 

*BI-RADS 4 Subcategories* 

BI-RADS 4 subcategory malignancy rates demonstrated appropriate risk stratification: 4A (32.7%), 4B (46.3%), and 4C (82.1%). These rates are higher than ACR-recommended ranges but consistent with other Indian studies, possibly reflecting population-specific factors, referral bias to tertiary centers, or interpretation patterns [12,13]. 

*BI-RADS 5 Lesions* 

The 91.8% malignancy rate for BI-RADS 5 lesions aligns well with the expected >95% range, validating the high specificity of this category for identifying lesions requiring immediate tissue sampling and oncological evaluation. 

Age-related considerations and epidemiological patterns 

The significant age-related increase in malignancy rates supports established epidemiological data and current screening guidelines emphasizing increased vigilance in older age groups. The peak incidence in the 40-60 years age group (51.1% of cases) aligns with global epidemiological data and Indian cancer registry reports [14]. 

The relatively higher proportion of malignancy in younger age groups compared to Western populations may reflect genetic predisposition, hormonal factors, or referral patterns in the Indian population. This finding has implications for screening strategies and clinical decision-making in younger women presenting with breast lumps. 

Clinical performance and diagnostic accuracy 

*High Sensitivity* 

The excellent sensitivity of 96.3% ensures minimal missed cancers, which is crucial for a screening and diagnostic tool. This high sensitivity provides confidence in using BI-RADS 4 and 5 categories as indicators for tissue sampling. 

Moderate Specificity 

The relatively lower specificity of 38.3% reflects the challenging nature of breast lesion differentiation and the tendency to err on the side of caution in suspicious cases. While this may result in some unnecessary biopsies, the high negative predictive value (91.1%) provides reassurance for BI-RADS 3 lesions managed with follow-up. 

*Clinical Decision Making* 

The strong correlation between BI-RADS categories and malignancy rates (p < 0.001) supports evidence-based clinical decision-making and validates current management algorithms recommending tissue sampling for BI-RADS 4 and 5 lesions.

Healthcare system and resource implications 

Tertiary Care Context 

As a 1100-bed tertiary referral center serving rural Karnataka and neighboring states, our institution provides comprehensive breast care including advanced imaging, multidisciplinary oncology services, and specialized pathology expertise. The study validates BI-RADS utility in this resource-appropriate setting, supporting its continued implementation in Indian healthcare systems. 

*Cost-Effectiveness Considerations* 

The high sensitivity and appropriate risk stratification support the cost-effectiveness of BI-RADS-guided management, reducing unnecessary procedures while ensuring appropriate cancer detection. The strong correlation enables confident clinical decision-making and optimal resource utilization. 

*Multimodal Imaging Integration* 

The comprehensive imaging approach utilizing mammography, ultrasonography, tomosynthesis, and selective MRI demonstrates the value of multimodal assessment in complex cases. The 41.4% utilization rate of tomosynthesis, particularly in dense breast evaluation, reflects evolving practice patterns and technology adoption in tertiary care settings.

Comparison with international literature 

Our findings align well with recent international studies while showing some population-specific variations. The 2025 prospective study by Ciurescu et al. from Romania reported comparable overall diagnostic performance with 93.8% sensitivity and 80.0% specificity, though our specificity was lower at 38.3% [5]. This difference may reflect variations in patient population, referral patterns, or interpretation thresholds. 

Khan et al. (2023) reported 44.9% positive predictive value for BI-RADS 4 lesions in a Pakistani cross-sectional study of 227 patients [6]. Our combined BI-RADS 4 subcategory analysis showed higher overall malignancy rates, possibly reflecting the tertiary referral nature of our institution and case complexity. A comprehensive comparison of our study's diagnostic performance with recent international and Indian literature is presented in Table 5

**Table 5:** Comparison with Published Literature PPV: Positive predictive value; NPV: negative predictive value

Study	Sample Size	Study Design	Sensitivity (%)	Specificity (%)	PPV (%)	NPV (%)	Accuracy (%)
Ciurescu et al. 2025 (Romania) [5]	67	Prospective	93.8	80	87.5	89	89.4
Khan et al. 2023 (Pakistan) [6]	227	Cross sectional	73.9	74.2	44.9	91	74
Lalchhanhimi et al. 2022 (India) [13]	80	Retrospective	86.7	99.4	94.1	96.4	89.5
Dariya et al. 2022 (India) [8]	60	Observational	91.7	88.9	84.6	94.1	90
Aziz et al. 2022 (Malaysia) [7]	316	Retrospective	94.5	43.1	63.9	88	68.8
Present Study (India)	215	Retrospective	96.3	38.3	61.2	91.1	67.4

Strengths

This study has several clear strengths. First, the sample size of 215 patients over two years is large enough to give reliable statistics and meaningful results. Second, every case had a confirmed histopathology report from biopsy or surgery, so the link between BI-RADS scores and final diagnosis is strong and trustworthy. Third, the imaging reflects real-life practice in a tertiary hospital, using digital mammography, ultrasound, tomosynthesis, and MRI when needed, so the results are relevant for similar centers. Fourth, experienced breast radiologists used the BI-RADS 5th edition, and difficult or mismatched cases were discussed in a multidisciplinary team, which improves the accuracy and consistency of reporting. Finally, the study provides detailed numbers for sensitivity, specificity, PPV, NPV, accuracy, odds ratio, and age-wise malignancy rates, making it easy for clinicians to understand how well BI-RADS performs in this setting.

Limitations

There are also some important limitations. Because the study is retrospective, it depends on existing records, which can introduce selection bias and missing information, and the reasons for doing imaging or biopsy cannot be fully controlled. The study included only adult patients (≥18 years), and findings may not be generalizable to the rare cases of breast malignancy in adolescent populations. It is from a single tertiary-care hospital, so the findings may not apply to smaller hospitals, different regions, or other healthcare systems. Follow-up for BI-RADS 3 cases was only to the minimum required period, so very late cancers in this group may have been missed, possibly underestimating their true risk. The study did not formally compare BI-RADS scores between different radiologists, so we do not know how reproducible the scoring is. As a referral center, the hospital likely sees more difficult and suspicious cases, which can explain the higher malignancy rates even in lower BI-RADS categories and the relatively low specificity. Lastly, the results reflect the South Indian population served by this hospital and may not directly match patterns seen in other ethnic groups or countries.

Future research directions

Artificial Intelligence Integration

Integration of artificial intelligence tools for automated BI-RADS assessment and decision support represents an emerging opportunity for enhanced diagnostic accuracy and consistency [15]. Machine learning algorithms could potentially improve standardization and reduce inter-observer variability [16].

Quantitative Imaging Biomarkers

Development and validation of quantitative imaging biomarkers including texture analysis, radiomics features, and perfusion parameters may enhance diagnostic accuracy and provide additional prognostic information [17].

Long-Term Outcome Studies

Prospective follow-up studies evaluating long-term outcomes for different BI-RADS categories, particularly category 3 lesions, would provide valuable data for management protocol optimization [18,19].

## Conclusions

This retrospective study of 215 patients at R.L. Jalappa Hospital, Kolar, validates the strong correlation between BI-RADS categorization and histopathological outcomes in breast lump evaluation. The progressive increase in malignancy rates from BI-RADS 3 (8.9%) to BI-RADS 5 (91.8%) confirms the effectiveness of the BI-RADS system for risk stratification in the Indian healthcare context. The diagnostic performance, particularly the high sensitivity of 96.3% and NPV of 91.1%, supports the use of BI-RADS as a reliable tool for guiding clinical decisions, including the recommendation for biopsy in categories 4 and 5.

These findings reinforce the clinical utility of the standardized BI-RADS lexicon in a tertiary care setting, contributing to evidence-based practice and improved patient management. While the specificity of 38.3% suggests potential for unnecessary biopsies, the primary goal of minimizing missed cancers is achieved. Future research should focus on prospective, multicenter studies to further refine BI-RADS application in diverse Indian populations and explore the integration of emerging technologies like artificial intelligence to enhance diagnostic precision and reduce inter-observer variability.
